# Markers for Major Complications at Day-One Postoperative in Fast-Track Metabolic Surgery: Updated Metabolic Checklist

**DOI:** 10.1007/s11695-023-06782-1

**Published:** 2023-08-23

**Authors:** J. W. H. ‘t Hart, R. Takken, C. R. C. Hogewoning, L. U. Biter, J. A. Apers, H. Zengerink, M. Dunkelgrün, C. Verhoef

**Affiliations:** 1 Department of Surgery, Franciscus Gasthuis & Vlietland, Kleiweg 500, 3045 PM Rotterdam, The Netherlands; 2 Department of Surgery, St. Antonius Hospital, Utrecht, The Netherlands; 3 Department of Surgery, Tulp Medisch Centrum, Zwijndrecht, The Netherlands; 4 Department of Surgery, Erasmus MC, Rotterdam, The Netherlands

**Keywords:** Enhanced recovery after bariatric surgery, Major complication, Safe discharge, Sleeve gastrectomy, Roux-en-Y gastric bypass, One-anastomosis gastric bypass

## Abstract

**Introduction:**

In fast-track metabolic surgery, the window to identify complications is narrow. Postoperative checklists can be useful tools in the decision-making of safe early discharge. The aim of this study was to evaluate the predictive value of a checklist used in metabolic surgery.

**Methods:**

Retrospective data from June 2018 to January 2021 was collected on all patients that underwent metabolic surgery in a high-volume bariatric hospital in the Netherlands. Patients without an available checklist were excluded. The primary outcome was major complications and the secondary outcomes were minor complications, readmission, and unplanned hospital visits within 30 days postoperatively.

**Results:**

Major complications within 30 days postoperatively occurred in 62/1589 (3.9%) of the total included patients. An advise against early discharge was significantly more seen in patients with major complications compared to those without major complications (90.3% versus 48.1%, *P* < 0.001, respectively), and a negative checklist (advice for discharge) had a negative predictive value of 99.2%. The area under the curve for the total checklist was 0.80 (*P* < 0.001). Using a cut-off value of ≥3 positive points, the sensitivity and specificity were 65% and 82%, respectively. Individual parameters from the checklist: oral intake, mobilization, calf pain, willingness for discharge, heart rate, drain (>30 ml/24 h), hemoglobin, and leukocytes count were also significantly different between groups.

**Conclusion:**

This checklist is a valuable tool to decide whether patients can be safely discharged early. Heart rate appeared to be the most predictive parameter for the development of major complications. Future studies should conduct prediction models to identify patients at risk for major complications.

**Graphical Abstract:**

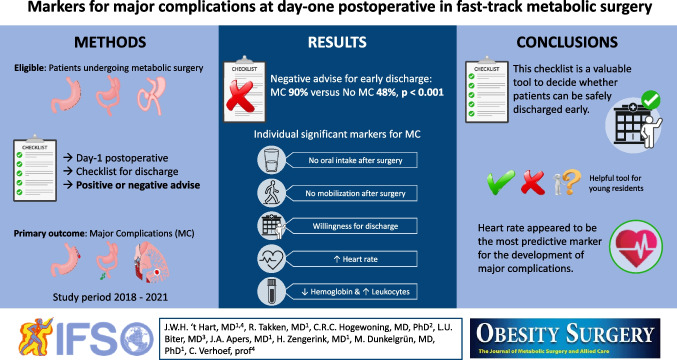

**Supplementary Information:**

The online version contains supplementary material available at 10.1007/s11695-023-06782-1.

## Introduction

Obesity is a growing global issue [1]. In patients with severe obesity, metabolic surgery has been proven the most effective long-term solution for weight reduction and improving comorbidities such as type 2 diabetes, hypertension, dyslipidemia, and the sleep apnea [2–5]. Despite many advantages of metabolic surgery, the procedure has also potential risk. In order to improve the patient’s outcome, the enhanced recovery after bariatric surgery (ERABS) protocol was developed. This protocol enables patients to be discharged at day one postoperatively [6, 7]. Some studies even suggest that same-day discharge after Roux-en-Y gastric bypass (RYGB) is safe [8]. This small window presents a challenge to observe major complications, especially in patients with severe obesity who are more at risk for complications such as hemorrhage, anastomotic leaks, stenosis, and thrombosis [9, 10].

Checklists are increasingly utilized to identify early complications following surgical treatments, and can be effective when properly deployed in preventing or reducing complications such as prolonged hospital stay, readmission, reoperation, and even mortality [11–14]. In 2017, van Mil et al. published a standardized postoperative checklist for metabolic surgery [15]. The patient’s willingness to be discharged home and a decrease in hemoglobin level were found to be significant predictors for complications in this study. Several studies evaluated hemoglobin level, heart rate, postoperative C-reactive protein (CRP), and leukocyte count as postoperative predictive parameters for complications after metabolic surgery, but there is no consensus on their predictive value [16–20]. In current practice, metabolic surgery is increasingly performed in daycare surgery and ward rounds are performed by young residents in many hospitals, to discharge patients home as safely as possible, a checklist can be a useful tool [21].

A useful checklist needs accurate cut-off points and unnecessary parameters should be omitted. The aim of this study was to evaluate the predictive value of the outcome of the checklist and its individual parameters. In addition, new cut-off values for linear variables from the checklist were determined.

## Methods

### Design and Data Collection

This single center retrospective cohort study includes all patients that underwent a laparoscopic sleeve gastrectomy (SG), RYGB, or one-anastomosis gastric bypass (OAGB) between June 2018 and January 2021. The choice of procedure was made by the surgeon and patient based on patient characteristics and preference. Patients were selected for metabolic surgery in accordance to IFSO criteria and excluded in case the checklist was not available [22]. Preoperatively at inclusion, a blood sample was obtained. Postoperatively, all patients were treated according to the ERABS protocol. The first day after surgery, the checklist was filled in by the surgical resident or physician assistant. Data was collected on baseline characteristics, checklist content, length of hospital stay, type of procedure, minor and major complications, hospital readmissions, and unplanned revisits to the emergency ward or the outpatient clinic and mortality.

### Postoperative Checklist

The postoperative checklist for metabolic surgery from the study published in 2017 by van Mil et al. was used. Calf pain was added to the checklist with the hypothesis of predicting venous embolisms. The study hospital is a high-volume center with approximately 900 metabolic procedures annually. Surgery was performed by four experienced metabolic surgeons. A visual analogue scale (VAS) was used for scoring pain. Only if tissue was prone to tearing or bleeding during surgery, a silicone wound drain Ch 30 + RO (Dispo Medical©) was placed to monitor potential bleeding or leakage. Table 1 shows the parameters and cut-off points. The postoperative checklist advised whether a patient could be safely discharged or should stay in the hospital for further observation or examinations. The final decision was made by the attending bariatric surgeon.

**Table 1:** Parameters of the postoperative checklist for metabolic surgery

Parameter	Score	Cut-off points
History		
VAS for pain	0–10	≥4
Nausea score	1–4	≥4
Ate liquid food?	Yes/no	No
Mobilizing?	Yes/no	No
Patient is willing to go home?	Yes/no	No
Physical examination		
Abdominal guarding?	Yes/no	Yes
Calf pain?	Yes/no	Yes
Heart rate		≥120 bpm
Oxygen saturation		≤90%
Drain production in 24 h		≥30 ml
Temperature		≥38°
Laboratory findings		
Hemoglobin decrease		≥1 mmol/L, or ≥1.6 g/dl
White blood cell count postoperative		≥14 × 10^9/L
C-reactive protein postoperative		≥79 mg/L
Total score maximum		13 points

*VAS* visual analogue scale, *bpm* beats per minute, *ml* milliliter, *mmol/L* millimol per liter, *mg/L* milligram per liter

### Outcomes

The occurrence of a major complication within 30 days of surgery was used as a primary outcome measure. Secondary outcomes were minor complications, readmission, and unplanned visits to the hospital within 30 days postoperatively. Complications were scored using the Clavien-Dindo (CD) classification [23].

### Statistical Analysis

Analyses were performed using SPSS (PASW) 28 software (SPSS Inc., Chicago, Illinois, USA). Patient outcomes were described as absolute number with percentage or median with interquartile range (IQR). The differences between patients with and without major complications, minor complications, readmission, and unplanned revisits were analyzed using the chi-squared test, Fisher exact, or Mann-Whitney *U* test when appropriate. Multinomial logistic regression analysis was used to estimate the relationship between the occurrence of major complications and the checklist outcomes, adjusting for baseline characteristics. Receiver operating characteristics (ROC) curves were constructed for linear checklist values (Table 1) on major complications. For all values with an area under the curve (AUC) greater than 0.70, the optimal cut-off value for predicting postoperative complications was determined. Results were evaluated at a significant threshold of *P* < 0.05 (two-sided).

## Results

### Baseline Characteristics

A total of 1593 patients were included between June 2018 and January 2021. Four patients were excluded because they underwent fundoplication surgery. Patient characteristics are shown in Table 2.

**Table 2:** Baseline characteristics in absolute numbers or median value, with its percentage or IQR

	Total (*n* = 1589)
Sex (female)	1248 (78.5%)
Age (years)	44 (33–53)
BMI (kg/m^2^)	39.9 (37.5–43.2)
Weight (kg)	113 (102.7–126.6)
Smoking	43 (2.7%)
Medical history	
Type 2 diabetes	270 (17.0%)
Hypertension	532 (33.5%)
Dyslipidemia	308 (19.4%)
OSAS	288 (18.1%)
GERD	318 (20.0%)
Abdominal surgery	628 (39.5%)
Cardiovascular disease	124 (7.8%)
Thromboembolic events	68 (4.3%)
COPD	71 (4.5%)
Anticoagulant use	148 (9.3%)
Immunosuppressive medication or disease	39 (2.5%)
Renal transplantation	4 (0.3%)
Dialysis	4 (0.3%)
Antidepressants use	77 (4.8%)
Characteristics procedure	
RYGB	551 (34.7%)
SG	871 (54.8%)
OAGB	167 (10.5%)
ASA score	
1	13 (0.8%)
2	145 (9.1%)
3	1311 (82.5%)
4	120 (7.6%)
Procedure time (minutes)	39 (32–47)

*IQR* interquartile range, *BMI* body mass index, *kg/m*^*2*^ kilograms per square meter, *OSAS* obstructive sleep apnea syndrome, *GERD* gastro-esophageal reflux disease, *COPD* chronic obstructive pulmonary disease, *RYGB* laparoscopic Roux-en-Y gastric bypass, *SG* laparoscopic sleeve gastrectomy, *OAGB* one-anastomosis gastric bypass, *ASA* American Society of Anesthesiologists

### Procedure Characteristics

The SG was most frequently performed 871/1589 (54.8%), followed by RYGB 551/1589 (34.7%) and the OAGB 167/1589 (10.5%). The median procedure time, recorded from first incision by the surgeon until the last suture, was 39 min (32–47), using the ERABS protocol [24].

### Complications

Major complications (hemorrhage, anastomotic leakage, perforation, and stenosis) occurred in 62/1589 (3.9%) patients. Baseline characteristics of patients were significantly different for age (*P* = 0.004), BMI (*P* = 0.038), hypertension (*P* = 0.011), dyslipidemia (*P* = 0.003), cardiovascular disease (*P* = 0.013), anticoagulant use (*P* = 0.006), and procedure time (*P* = 0.028) (Table 3). The most frequent major complication observed was hemorrhage (CD ≥ 3), occurring in 38/62 (61%) of the patients experiencing complications and 38/1589 (2.4%) overall. Hemorrhage was diagnosed based on a significant decrease in hemoglobin levels and clinical symptoms in 24/38 (63.2), or by a CT scan 14/38 (36.8%). Reoperation was performed in 31/38 (81.6%) patients, gastroscopy in 4/38 (10.5%) patients, and 3/38 (7.9%) patients received packed red blood cells without requiring further intervention.

**Table 3:** Baseline characteristics between patients with no and major complications in absolute numbers or median value with its percentage or IQR

	No major complications (*n* = 1527)	Major complications (*n* = 62)	*P* value
Sex (female)	1202 (78.7%)	46 (74.2%)	*P* = 0.395^a^
Age (years)	44 (32–53)	48 (43–56)	*P* = 0.004^c^
BMI (kg/m^2^)	39.9 (37.5–43.2)	38.9 (36.7–40.9)	*P* = 0.038^c^
Weight (kg)	113.1 (102.8–126.8)	110.7 (101.8–124.2)	*P* = 0.600^c^
Smoking	41 (2.7%)	2 (3.2%)	*P* = 0.683^b^
Medical history			
Type 2 diabetes	259 (17.0%)	11 (17.7%)	*P* = 0.873^a^
Hypertension	502 (32.9%)	30 (48.4%)	*P* = 0.011^a^
Dyslipidemia	287 (18.8%)	21 (33.9%)	*P* = 0.003^a^
OSAS	274 (17.9%)	14 (22.6%)	*P* = 0.353^a^
GERD	305 (20.0%)	13 (21.0%)	*P* = 0.848^a^
Abdominal surgery	604 (39.6%)	24 (38.7%)	*P* = 0.894^a^
Cardiovascular disease	114 (7.5%)	10 (16.1%)	*P* = 0.013^a^
Thromboembolic events	65 (4.3%)	3 (4.8%)	*P* = 0.746^b^
COPD	69 (4.5%)	2 (3.2%)	*P* = 1.000^b^
Anticoagulant use	136 (8.9%)	12 (19.4%)	*P* = 0.006^a^
Immunosuppressive medication/disease	35 (2.3%)	4 (6.5%)	*P* = 0.062^b^
Renal transplantation	4 (0.3%)	0 (0.0%)	*P* = 1.000^b^
Dialysis	74 (4.8%)	3 (4.8%)	*P* = 1.000^b^
Antidepressants use	3 (0.2%)	1 (1.6%)	*P* = 0.147^b^
Characteristics procedure			
Procedure type			*P* = 0.246^a^
SG	842 (55.1%)	29 (46.8%)	
RYGB	528 (34.6%)	23 (37.1%)	
OAGB	157 (10.3%)	10 (16.1%)	
ASA score			*P* = 0.896^a^
1	12 (0.8%)	1 (1.6%)	
2	140 (9.2%)	5 (8.1%)	
3	1260 (82.5%)	51 (82.3%)	
4	115 (7.5%)	5 (8.1%)	
Procedure time (minutes)	39 (32–46)	41 (34–52)	*P* = 0.028^c^

*IQR* interquartile range, *BMI* body mass index, *kg/m*^*2*^ kilograms per square meter, *OSAS* obstructive sleep apnea syndrome, *GERD* gastro-esophageal reflux disease, *COPD* chronic obstructive pulmonary disease, *SG* laparoscopic sleeve gastrectomy, *RYGB* laparoscopic Roux-en-Y gastric bypass, *OAGB* one-anastomosis gastric bypass, *ASA* American Society of Anesthesiologists. ^a^Chi-squared test, ^b^Fisher exact, ^c^Mann-Whitney *U* test

### Checklist Outcome

Patients with major complications were significantly more likely to get a negative discharge advice following the checklist, requiring consultation with the metabolic surgeon when compared to patients without major complications (90.3% versus 48.1%, *P* < 0.001, respectively). Of all parameters included in the checklist, oral intake, mobilization, calf pain, willingness for discharge, heart rate, drain > 30 ml/24 h, hemoglobin postoperative, hemoglobin decrease, and leukocytes were significantly different between groups. After correcting for confounders, all significant parameters remained significant (Table 4). In 38 (2.4%) patients with a hemorrhage (CD ≥ 3), 37 (97.4%) patients received a negative advice. One patient was initially discharged with a positive advise (showing no signs), but later readmitted. After correction for covariables, significant differences were observed in nausea, oral intake, mobilization, willingness for discharge, heart rate, hemoglobin postoperative, hemoglobin decrease, and leukocytes. In 39 (2.5%) patients with minor hemorrhage (CD ≤ 2), 36 (92.3%) received a negative advise. Supplementary file 1 displays the difference between no hemorrhage, minor, and major hemorrhage. Leakage within 30 days was seen in nine (0.6%) patients, and after correcting for confounders, only CRP was significantly different in these patients (median 78 versus 19, *P* = 0.003, respectively).

**Table 4:** Checklist outcome between patients with no and major complications in absolute numbers or median value with its percentage or IQR

	No complication (*n* = 1527)	Major complication (*n* = 62)	*P* value
Hospitalization time (hours)	28.8 (27–32)	90.5 (45–120)	*P* < 0.001^**c**^
Nausea scale			*P* = 0.070^a^
No nausea	1073 (70.8%)	35 (60.3%)	
Nausea	310 (20.7%)	15 (25.9%)	
Gagging	30 (2.0%)	5 (8.6%)	
Vomiting	82 (5.5%)	3 (5.2%)	
Oral intake			
No	99 (6.6%)	16 (27.6%)	*P* < 0.001^a^
Mobilizing			
No	12 (0.8%)	9 (14.8%)	*P* < 0.001^a^
Calf pain			
Yes	68 (4.9%)	6 (12.2%)	*P* < 0.035^b^
Willingness for discharge			
No	168 (11.3%)	27 (45.0%)	*P* < 0.001^a^
Temperature (°C)	37.1 (36.8–37.4)	37 (36.8–37.5)	*P* = 0.909^c^
Heart rate (bpm)	77 (69–86)	91 (78–105)	*P* < 0.001^c^
Oxygen saturation (%)	96 (95–98)	96 (95–97)	*P* = 0.231^c^
Drain production			*P* = 0.001^b^
No drain	1509 (98.8%)	56 (91.8%)	
<30 ml/24 h	7 (0.5%)	1 (1.6%)	
>30 ml/24 h	12 (0.8%)	4 (6.6%)	
VAS for pain ≥ 4			*P* = 0.173^a^
Hemoglobin (mmol/L)	627 (41.3%)	31 (50.0%)	*P* < 0.001^c^
Hemoglobin decrease (mmol/L)	8.1 (7.6–8.6)	7.5 (6.9–8.2)	*P* < 0.001^c^
Leukocyte count (×10^9^/L)	−0.5 (−0.9 to −0.2)	−1.2 (−1.9 to −0.4)	*P* = 0.004^c^
CRP (mg/L)	11.5 (9.7–13.5)	12.8 (10.6–14.9)	*P* = 0.060^c^
Abdominal distension	19 (12–30)	24 (12–44)	*P* = 1.000^b^
Advice checklist	4 (0.3%)	0 (0.0%)	
No discharge	735 (48.1%)	57 (91.9%)	*P* < 0.001^a^

*IQR* interquartile range, *°C* Celsius, *bpm* beats per minute, *mmol/L* millimol per liter, *VAS* visual analogue scale, *CRP* C-reactive protein, *mg/L* milligram per liter. ^a^Chi-squared test, ^b^Fisher exact, ^c^Mann-Whitney *U* test

### Positive and Negative Predictive Values

For the development of a major complication, the postoperative checklist for metabolic surgery had a positive predictive value of 7.1% and a negative predictive value of 99.2%. Patients with a negative advise for the checklist had a significant higher chance of a complication with an odd ratio of 10.1 ([95% CI 4.3 to 23.5], *P* < 0.001). Of the total population (no or major complications) with a positive checklist (advice against discharge), the true positive rate was 91.9% and the true negative rate was 48.1%.

### ROC Analysis

**Fig. 1:**
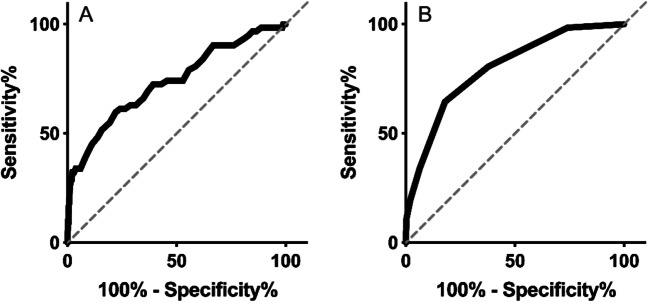
**A** ROC curves of postoperative heart rate and **B** of total score checklist as markers for major complications in patients after metabolic surgery: Analysis demonstrated an area under the curve of 0.74 (*P* < 0.001) and 0.79 (*P* < 0.001) for total checklist score. Abbreviation: ROC curve, receiver operating characteristics curve

ROC analyses were conducted for significant different outcomes between the group with and without major complications. The AUC according to the ROC analysis for the total checklist was 0.80 (*P* < 0.001). With a cut-off value of ≥3 positive points, the sensitivity is 65% and the specificity is 82%. The AUC according to the ROC analysis for heart rate was 0.75 (*P* < 0.001). The cut-off point of 80.5 bpm for heart rate had the highest combination of sensitivity of 74% and a specificity of 61%. Both ROC curves are shown in Fig. 1A–B. The AUC for hemoglobin, hemoglobin decrease, and leukocyte count were significant for major complications; however, these results did not meet the criteria of an AUC of 0.7.

### Minor Complications

Minor complications occurred in 133 (8.4%) patients. Patients with minor complications were significantly more likely to have a negative discharge advice from the checklist, requiring consultation of a metabolic surgeon when compared to patients without complications (60.9% versus 48.8%, *P* = 0.008, respectively). The nausea scale (*P* = 0.032), oral intake (*P* < 0.001), willingness for discharge (*P* < 0.001), VAS score (*P* < 0.001), hemoglobin postoperative (*P* < 0.001), hemoglobin decrease (*P* = 0.027), and postoperative CRP (*P* = 0.002) were significantly different between the groups. Of minor complications (no or minor complications) with a positive checklist (advice against discharge), the true positive rate was 60.8% and the true negative rate was 51.2%.

### Readmissions and Unplanned Revisits

In patients with a negative advise to go home, 33/791 (4.2%) patients were readmitted in comparison to 38/798 (4.8%) patients with a positive advise *P* = 0.569. In patients with a negative advise to go home, 100/791 (12.6%) patients had unplanned revisits in comparison to 77/798 (9.6%) patients with a positive advise *P* = 0.058. Seventy-one (4.5%) patients were readmitted to the hospital with a significant decrease in hemoglobin (*P* = 0.030) and increase of CRP (*P* = 0.008). Major complications occurred in 30% of the readmitted patients and minor complications in 63%. One hundred seventy-seven (11.1%) patients had unplanned visits to the outpatient clinic or emergency room, and were found to have significant differences in willingness to be discharged (*P* < 0.001), VAS score for pain (*P* < 0.001), postoperative hemoglobin (*P* = 0.024), and CRP level (*P* = 0.006) (Table 5). For the readmission category and a positive checklist (advice against discharge), the true positive rate was 47.9% and the true negative rate was 50.0%. Regarding unplanned revisits with a positive checklist (advice against discharge), the true positive rate was 57.0% and the true negative rate was 51.1%.

**Table 5:** Checklist outcome in patients with minor complications, readmission, and unplanned revisit in absolute numbers with percentage or median value with its IQR

	Minor complication (*n* = 133)	*P* value	Readmissions (*n* = 71)	*P* value	Unplanned revisits (*n* = 177)	*P* value
Nausea scale	85 (63.9%)	*P* = 0.032^a^		*P* = 0.353^a^		*P* = 0.090^a^
No nausea	36 (27%)		44 (62%)		110 (62.1%)	
Nausea	1 (0.7%)		18 (25.4%)		45 (25.4%)	
Gagging	11 (8.3%)		3 (4.2%)		4 (2.3%)	
Vomiting	19 (14.3%)		5 (7%)		14 (7.9%)	
Oral intake (no)	4 (3.0%)	*P* < 0.001^a^	6 (8.5%)	*P* = 0.691^a^	18 (10.2%)	*P* = 0.104^a^
Mobilizing (no)	7 (5.3%)	*P* = 0.069^b^	0 (0.0%)	*P* = 0.621^b^	1 (0.6%)	*P* = 0.722^b^
Calf pain (yes)	30 (22.6%)	*P* = 0.482^a^	6 (8.4%)	*P* = 0.142^a^	12 (6.8%)	*P* = 0.144^a^
Willingness for discharge (no)	37.2 (36.8–37.5)	*P* < 0.001^a^	14 (19.7%)	*P* = 0.051^a^	37 (20.9%)	*P* < 0.001^a^
Temperature (°C)	77 (69–89)	*P* = 0.602^c^	37 (36.8–37.4)	*P* = 0.368^c^	37.1 (36.8–37.4)	*P* = 0.572^c^
Heart rate (bpm)	96 (95–97)	*P* = 0.952^c^	78 (70–89)	*P* = 0.452^c^	77 (69–88)	*P* = 0.727^c^
Oxygen saturation (%)		*P* = 0.123^c^	96 (95–98)	*P* = 0.955^c^	96 (95–97)	*P* = 0.306^c^
Drain production	130 (97.7%)	*P* = 0.763^a^		*P* = 0.246^a^		*P* = 0.618^a^
No drain	1 (0.8%)		69 (97.2%)		173 (97.7%)	
<30 ml/24 h	2 (1.5%)		0 (0.0%)		1 (0.6%)	
>30 ml/24 h	75 (56.4%)		2 (2.8%)		3 (1.7%)	
VAS for pain ≥4	7.9 (7.3–8.4)	*P* < 0.001^a^	37 (52.1%)	*P* = 0.071^a^	101 (57.4%)	*P* < 0.001^c^
Hemoglobin (mmol/L)	0.6 (0.3–1)	*P* < 0.001^c^	8.1 (7.4–8.5)	*P* = 0.625^c^	8 (7.4–8.5)	*P* = 0.024^c^
Hemoglobin decrease (mmol/L)		*P* = 0.027^c^	0.4 (0.0–0.7)	*P* = 0.030^c^	0.5 (0.2–0.9)	*P* = 0.813^c^
Leukocyte count (×10^9^/L)	11.3 (9.4–13.3)	*P* = 0.251^c^	10.9 (9.3–13.2)	*P* = 0.193^c^	11.5 (9.5–13.4)	*P* = 0.468^c^
CRP (mg/L)	23 (14–38)	*P* = 0.002^c^	23 (15–43)	*P* = 0.008^c^	23 (14–37.5)	*P* = 0.006^c^
Abdominal distension	1 (0.8%)	*P* = 0.296^b^	1 (1.4%)	*P* = 0.168^b^	1 (0.6%)	*P* = 0.377^b^
Advice checklist (no discharge)	81 (60.9%)	*P* = 0.007^a^	33 (46.5%)	*P* = 0.569^a^	100 (56.5%)	*P* = 0.058^a^

*IQR* interquartile range, *°C* Celsius, *bpm* beats per minute, *mmol/L* millimol per liter, *VAS* visual analogue scale, *CRP* C-reactive protein, *mg/L* milligram per liter. ^a^Chi-squared test. ^b^Fisher exact. ^c^Mann-Whitney *U* test

## Discussion

The narrow time frame and frequency of major complications for patients with extreme obesity following weight loss surgery highlights the importance of implementing a checklist to facilitate safe discharge. The aim of this study was to re-evaluate and validate the predictive value of a postoperative checklist and to evaluate individual parameters for major complications after metabolic surgery. This study showed that our postoperative checklist was significantly more likely to give a negative advice in patients developing a major complication, with heart rate being the most predictive individual parameter.

In 90.3% of patients with major complication, the checklist gave a negative discharge advice. However, the positive predicted value of the checklist was 7.1%, meaning large numbers of reevaluations could be avoided. Nonetheless, the negative predicted value was 99.2%, meaning most patients discharged home on the first postoperative day did not develop a major complication. The possible overtreatment outweighs the safeness of discharge [15].

In contradiction to the pilot study, not only the willingness to be discharged on day one postoperatively but also oral intake, ability to mobilize, and calf pain were significantly different between groups. When patients are unable to mobilize or eat, even after guidance of nurses according to the ERABS protocol, this may indicate a complication [6]. Calf pain was added to the checklist, as a potential predictor of venous thrombotic event (VTE) of the lower leg and could prevent the development of pulmonary embolism with high mortality [25]. Although this parameter was significantly higher, only 2 patients developed VTE, making the utility of this parameter questionable.

The most common major complication was hemorrhage 38/62 (61%) and 38/1589 (2.4%) of the total included patients. After metabolic surgery increase in heart rate is one of the first symptoms in patients with hemorrhage [16, 26, 27]. In 38 patients with hemorrhage from this study, the same trend was observed (97 bpm versus 78 bpm, *P* < 0.001; delta 19 bpm versus −0.2 bpm, *P* < 0.001, respectively). A heart rate of 87 bpm was found to have the best combination of sensitivity and specificity. A heart rate of 87 bpm is clinically relatively normal. As a result, more patients receive a negative advise, even if there are no complications which is not preferable. In our study, we observed also a significant difference in postoperative hemoglobin and delta-hemoglobin between patients with and without hemorrhage, which is consistent with earlier research on postoperative hemorrhage after metabolic surgery [16, 28]. Fecso et al. suggested a 2 mmol/L drop in hemoglobin as an indicator of intervention [16]. In our study, the identified cut-off value for delta-hemoglobin was 1.1 mmol/L, with a sensitivity of 87% and a specificity of 87%, and an AUC of 0.923 (*P* < 0.001), respectively. However, these results should be interpreted with caution due to the small sample size, and no definitive recommendations can be made.

Fever and peritonitis are not specific symptoms for hemorrhage; one would rather expect this for anastomotic leakage [29]. However, the group of patients with leakage was too small 9/1589 (0.6%) to achieve significance in temperature and abdominal distension. In addition, high CRP levels may be useful in identifying patients with anastomotic leakage [17, 18, 20]. Median CRP level in the group was 78 mg/L, almost identical to the recommended cut-off value of >79 mg/L. However, the results of this subanalyses should be interpreted with caution, no recommendation could be given.

Drainage might reflect the surgeon’s intraoperative evaluation and anticipation of complications [30]. In the current study, >30 cc/24 h drain output was significantly higher in major complications. However, among 24 patients with a drain, 5 had complications. The uncertain utility of drain output in predicting complications has led to a decrease in drain usage percentages, which is also observed in our center [30, 31].

In the minor complication group, most patients had problems with toleration of oral intake, which explained that nausea, oral intake, and willingness of discharge had a significantly more negative outcome in the minor complication group. For readmission and unplanned revisits, the checklist was not predictive. After assessment by the attending surgeon, some patients were allowed to go home regardless of the negative advice of the checklist. For patients with no willingness for discharge, a high VAS score, or a high CRP without any other findings suggestive of complications, more guidance in the postoperative process is imperative in future practice to prevent unnecessary readmissions or revisits.

Although this study had sufficiently large numbers, the COVID-19 pandemic resulted in fewer metabolic procedures than expected. Second, a fluctuation was observed in the time frame when vital signs were collected during the nurses’ round, ranging from early to late in the morning. This may have affected the outcome. Better communication is necessary between physicians and nurses for measurement of vital parameters and time of ward rounds. Thirdly, most AUCs were too small to calculate new cut-off values. Parameters were assessed individually, while some parameters may be related. It is worth noting that four patients had revision surgery, none had a major complication, and only one had a minor complication. Including these cases reflected real-clinical practice, as the checklist is used for all bariatric patients. A larger sample size in this subgroup would have provided more insights into the risk of complications in revision surgery [32]. For a future study, a prediction model may be even more specific for predicting complications. The neutrophil-to-lymphocyte ratio (NLR) and increase in heart rate were not included in this study, but have shown to be of great value [33, 34]. To examine the new checklist with cut-off values and other additional parameters, we advise it should be externally validated.

In conclusion, the checklist may be a useful tool to identify the patients that can be safely discharged home. Heart rate seems the most predictive parameter for major complications. We recommend that a predictive model of the modified checklist with additional parameters, such as increase in heart rate and NLR to identify patients at risks for major complications, be externally validated.

### Supplementary Information

ESM 1
