# Supplementary material for: Markers for Major Complications at Day-One Postoperative in Fast-Track Metabolic Surgery: Updated Metabolic Checklist
Source: Obes Surg. 2023 Aug 23;33(10):3008–16. doi: 10.1007/s11695-023-06782-1 (PMC10514089; doi:10.1007/s11695-023-06782-1)
Supplement: Supplementary file 1 — ESM 1 [file 11695_2023_6782_MOESM1_ESM.pdf]

Supplementary file 1

Sub-analyses for patients with major hemorrhage:

The most frequent major complication observed was hemorrhage (CD ≥ 3), occurring in 38/62 (61%) of the patients experiencing complications and 38/1589 (2.4%) overall. Hemorrhage was diagnosed based on a significant decrease in hemoglobin levels and clinical symptoms in 24/38 (63.2), or by a CT-scan 14/38 (36.8%). Reoperation was performed in 31/38 (81.6%) patients, gastroscopy in 4/38 (10.5%) patients, and 3/38 (7.9%) patients received packed red blood cells without requiring further intervention.

In 38 (2.4%) patients with a hemorrhage (CD ≥ 3), 37 (97.4%) patients received a negative advice. One patients was initially discharged with a positive advise (showing no signs), but later readmitted. After correction for covariables, significant differences were observed in, nausea, oral intake, mobilization, willingness for discharge, heart rate, hemoglobin postoperative, hemoglobin decrease, and leukocytes between patients with and without major hemorrhage (Table 1).

Table 1 Checklist outcome between patients with and without major hemorrhage

	No Hemorrhage (n= 1522)	Hemorrhage (n=38)	*P* value	Adjusted *P*-value
Hospitalization time (hours)	28.9 (27-32)	100 (76-124)	***P* < 0.001**[c]	*P* = 0.230[d]
Nausea scale			***P* < 0.001**[a]	***P* < 0.001**[e]
No nausea	1087 (71.6%)	20 (57.1%)		
Nausea	317 (20.9%)	8 (22.9%)		
Gagging	30 (2.0%)	6 (17.1%)		
Vomiting	84 (5.5%)	1 (2.9%)		
Oral intake			***P* < 0.001**[a]	***P* < 0.001**[e]
No	105 (6.9%)	10 (29.4%)		
Mobilizing			***P* < 0.001**[a]	***P* < 0.001**[e]
No	12 (0.8%)	9 (24.3%)		
Calf pain			*P* = 0.143[b]	*P* = 0.201[e]
Yes	71 (5.0%)	3 (11.5%)		
Willingness for discharge			***P* < 0.001**[a]	***P* < 0.001**[e]
No	176 (11.7%)	19 (52.8%)		
Temperature (°C)	37.1 (36.8-37.4)	37 (36.7-37.5)	*P* = 0.773[c]	*P* = 0.773[d]
Heart rate (bpm)	77 (69-86)	95 (86-108)	***P* < 0.001**[c]	***P* < 0.001**[d]
Oxygen saturation (%)	96 (95-98)	96 (95-97)	*P* = 0.274[c]	*P* = 0.274[d]
Drain production			***P* < 0.001**[b]	*P* = 0.103[e]
No drain	1531 (98.7%)	34 (89.5%)		
<30 ml/24hour	7 (0.5%)	1 (2.6%)		
>30 ml/24hour	13 (0.8%)	3 (7.9%)		
VAS for pain ≥4	641 (41.6%)	19 (50.0%)	*P* = 0.298[a]	*P* = 0.298[e]
Hemoglobin (mmol/l)	8.1 (7.6-8.6)	7.2 (6.6-7.8)	***P* < 0.001**[c]	***P* < 0.001**[d]
Hemoglobin decrease (mmol/l)	-0.5 (-0.9 to -0.2)	-1.7 (-2.3 to -1.3)	***P* < 0.001**[c]	***P* < 0.001**[d]
Leukocyte count (x10^9/l)	11.5 (9.7-13.5)	13.0 (11.1-15.4)	***P* < 0.001**[c]	***P* < 0.001**[d]
CRP (mg/l)	19 (12-30)	18 (11-36)	*P* = 0.803[c]	*P* = 0.803[d]
Abdominal distension	4 (0.3%)	0 (0.0%)	*P* = 1.000[b]	*P* = 1.000[e]
Advice checklist			***P* < 0.001**[a]	***P* < 0.001**[e]
No discharge	755 (48.7%)	37 (97.4%)		

In absolute numbers with percentage or median value with IQR. Adjusted *P*-values were corrected for covariables. *IQR* interquartile range, *°C* Celsius*, bpm* beats per minute, *mmol/L* millimol per liter, *VAS* Visual Analogue Scale, *CRP* C-reactive protein, *mg/L* milligram per liter. [a] Chi-squared test, [b] Fisher exact, [c] Mann-Whitney U test, [d] linear regression analyses, [e] logistic regression analyses.

Sub-analyses for patients with minor and major hemorrhage:

In 39 (2.5%) patients with minor hemorrhage (CD ≤ 2), 36 (92.3%) received a negative advise. Table 2 displays the difference between no hemorrhage, minor, and major hemorrhage.

Nausea, oral intake, oral intake, mobilization, willingness for discharge, heart rate, drain production, hemoglobin postoperative, hemoglobin decrease, and leukocytes were significant differences between patients with and without major hemorrhage (Table 2).

Table 2 Checklist outcome between patients without and with minor or major hemorrhage

	No Hemorrhage (n= 1510)	Minor Hemorrhage (n=39)	Hemorrhage (n=38)	*P* value
Hospitalization time (hours)	28.9 (27-32)	28.9 (27-32)	100 (76-124)	***P* < 0.001**[b]
Nausea scale				***P* < 0.001**[a]
No nausea	1065 (71.9%)	22 (61.1%)	20 (57.1%)	
Nausea	306 (20.6%)	11 (30.6%)	8 (22.9%)	
Gagging	30 (2.0%)	0 (0.0%)	6 (17.1%)	
Vomiting	81 (5.5%)	3 (8.3%)	1 (2.9%)	
Oral intake				***P* < 0.001**[a]
No	94 (6.3%)	11 (28.9%)	10 (29.4%)	
Mobilizing				***P* < 0.001**[a]
No	9 (0.6%)	3 (8.1%)	9 (24.3%)	
Calf pain				*P* = 0.264[a]
Yes	69 (4.9%)	2 (7.7%)	3 (11.5%)	
Willingness for discharge				***P* < 0.001**[a]
No	167 (11.3%)	9 (28.1%)	19 (52.8%)	
Temperature (°C)	37.1 (0.5)	37.1 (0.5)	37.1 (0.5)	*P* = 0.948[c]
Heart rate (bpm)	77.5 (12.2)	80.3 (17.1)	97.0 (16.6)	***P* < 0.001**[c]
Oxygen saturation (%)	96.4 (1.8)	96.0 (1.7)	96.0 (2.2)	*P* = 0.145[c]
Drain production				***P* < 0.001**[a]
No drain	1491 (98.7%)	39 (100%)	34 (89.5%)	
<30 ml/24hour	7 (0.5%)	0 (0.0%)	1 (2.6%)	
>30 ml/24hour	13 (0.9%)	0 (0.0%)	3 (7.9%)	
VAS for pain ≥4	617 (41.1%)	24 (61.5%)	19 (50.0%)	*P* = 0.022[a]
Hemoglobin (mmol/l)	8.1 (0.7)	7.1 (1.0)	7.2 (0.9)	***P* < 0.001**[b]
Hemoglobin decrease (mmol/l)	-0.5 (-0.5)	-1.6 (-0.5)	-1.7 (-0.7)	***P* < 0.001**[b]
Leukocyte count (x10^9/l)	11.8 (2.9)	10.9 (2.8)	13.8 (3.8)	***P* < 0.001**[b]
CRP (mg/l)	25.5 (22.0)	31.3 (20.8)	24.6 (19.3)	*P* = 0.803[b]
Abdominal distension	4 (0.3%)	0 (0.0%)	0 (0.0%)	*P* = 0.904[a]
Advice checklist				***P* < 0.001**[a]
No discharge	718 (47.5%)	36 (92.3%)	37 (97.4%)	

In absolute numbers with percentage or median value with IQR. *IQR* interquartile range, *°C* Celsius*, bpm* beats per minute, *mmol/L* millimol per liter, *VAS* Visual Analogue Scale, *CRP* C-reactive protein, *mg/L* milligram per liter. [a] Chi-squared test, [b] one-way Anova.
